# Complete Genome Sequence of Peptacetobacter (Clostridium) hiranonis Strain DGF055142, Isolated from Dog Feces from Flagstaff, Arizona, USA, 2019

**DOI:** 10.1128/MRA.00067-21

**Published:** 2021-03-04

**Authors:** Nathan E. Stone, Amalee E. Nunnally, Chandler C. Roe, Heidie M. Hornstra, David M. Wagner, Jason W. Sahl

**Affiliations:** a Pathogen and Microbiome Institute, Northern Arizona University, Flagstaff, Arizona, USA; b Department of Biological Sciences, Northern Arizona University, Flagstaff, Arizona, USA; University of Maryland School of Medicine

## Abstract

A single-chromosome closed genome of Peptacetobacter (Clostridium) hiranonis strain DGF055142 was generated using Illumina MiSeq short reads paired with Oxford Nanopore MinION long reads. This isolate was obtained from a canine in Flagstaff, Arizona, in 2019. *Peptacetobacter* (*C.*) *hiranonis* was hypothesized to contribute to canine Clostridium difficile infection resistance.

## ANNOUNCEMENT

Peptacetobacter (Clostridium) hiranonis is a normal component of healthy canine guts (1–3) and performs primary to secondary bile acid conversion via 7α-dehydoxylation (4, 5). Secondary bile acids have been shown to inhibit Clostridium difficile growth *in vitro* (6, 7), and the presence of *P.* (*C.*) *hiranonis* in canine guts has been hypothesized to contribute to resistance to C. difficile infection (1).

A live culture of *P.* (*C.*) *hiranonis* was isolated from feces obtained from a healthy 2-year-old Alaskan Klee Kai canine that was previously determined positive for *P.* (*C.*) *hiranonis* DNA (1, 8). Upon deposit, the sample was transferred to an anaerobic chamber (Coy Labs). A 10-μl loopful of sample was homogenized with 200 μl 1× sterile phosphate-buffered saline (PBS) and plated onto prereduced brain heart infusion salt (BHIS) agar plates supplemented with 2 μM hemin, 4 mM l-cysteine, and 2 mM taurocholic acid for 48 h at 37°C under anaerobic conditions. During incubation, the sample was confirmed to harbor *P.* (*C.*) *hiranonis* DNA by species-specific PCR (1, 8). Isolation streaks were performed on 20 colonies with *Clostridium*-like morphologies and incubated for 48 h. DNA was extracted from subcolonies using a 5% Chelex 100 heat soak method (9–11), and *P.* (*C.*) *hiranonis* PCR was conducted (1, 8); 16/20 were positive but not pure. Purification continued until two isolates were obtained. Isolates were propagated as lawns, and −80°C frozen stocks were prepared in 20% glycerol. Simultaneously, genomic DNA (gDNA) was extracted using Qiagen kits and prepped for whole-genome sequencing (WGS) on an Illumina MiSeq instrument (12, 13). One isolate (DGF055142) was pure as determined during WGS analysis [only *P.* (*C.*) *hiranonis* reads were identified] and prepared for long-read sequencing by adjusting a bacterial suspension to a 1.0 McFarland turbidity standard (Remel); a lawn was created and incubated at 37°C for 24 h. High-molecular-weight (HMW) gDNA was extracted using the Quick-DNA HMW MagBead kit (Zymo) and assessed for quality using a standard genomic 50-kb fragment analyzer kit (Agilent) to ensure mean DNA fragments of >60,000 kb. Additionally, *A*_260_/*A*_230_ and *A*_260_/*A*_280_ ratios were assessed using NanoDrop technology (Thermo Fisher) to confirm MinION suitability, and the DNA concentration was determined using a Qubit device (Thermo Fisher). Libraries were prepared using an SQK-LSK109 1D ligation gDNA kit with the native barcoding gDNA kit (Oxford Nanopore). Libraries were loaded onto an R9/R9.4 flow cell, and MinION sequencing was performed for 60 h using MinKNOW software; base calling was performed with Guppy v3.22 (Oxford Nanopore) using the 9.4.1_450bps_hac workflow.

Illumina reads were trimmed with bbduk.sh v38.86 (https://sourceforge.net/projects/bbmap/), MinION reads (total, 232,473; *N*_50_, 17,946) were trimmed with Porechop v0.2.4 (https://github.com/rrwick/Porechop), and a hybrid assembly was created with Unicycler v0.4.8 (14). The final assembly was polished using Pilon v1.23 (15) until no more corrections could be made and then was annotated with the NCBI PGAP pipeline (16). The depth and breadth of coverage were calculated by aligning sequence reads against the assembly with minimap2 v2.17 (17) and then calling the per-base coverage with SAMtools v1.10 (18). Default parameters were used for all software.

A single contig assembly was generated (2,534,695 bp; G+C content, 31.35%; 2,220 coding DNA sequences [CDSs]). Other assembly statistics are as follows: average depths of coverage, 116× (Illumina) and 609× (MinION); median depths of coverage, 121× (Illumina) and 612× (MinION); standard deviation (SD) depths of coverage, 17× (Illumina) and 101× (MinION); breadths of coverage (>10×), 99.99% (Illumina) and 99.72% (MinION); genome size, 2,534,695 bp; number of contigs, 1; number of CDSs, 2,220; and G+C content, 31.35%.

### Data availability.

All sequence data were deposited in NCBI GenBank under BioProject accession number PRJNA688511 and SRA number SRP299691. The completed genome assembly can be found under GenBank accession number CP066811.
